# Annual Report of the Royal Veterinary Institute at Vienna, for the Year 1883

**Published:** 1884-10

**Authors:** 


Annual Report of the Royal Veterinary Institute at Vienna, for the Year 1883.—The *Oesterreichische Vierteljahresschrift für Veterinairkünde* (Austrian Quarterly Journal of Veterinary Medicine) for the second quarter of the year 1884 is a very interesting and instructive number in that it demonstrates not only what the school has been doing during the past year, but also how the government helps advance the cause of veterinary medicine. In this number is a report from two of the teachers of the school that, during their last summer's vacation were sent by the government to inspect and report upon some of the largest breeding establishments of Germany and Hungary, also to visit the veterinary schools at Dresden and Berlin.

The Veterinary School at Vienna has two directors, a military "commandant," and a Director of Instruction; the latter position being at present occupied by Prof. Dr. Franz Müller.

The whole number of students during the year was 422, of whom 158 were military and 264 civil; 40 candidates presented themselves for examination, of whom 9 passed very satisfactorily and 28 as satisfactory; the others failed. The Library contains 10,638 volumes.

The whole number of animals treated in the hospital for large animals was 2,262, of which 2,255 were horses. The months of May and June showed the most sickness, the hospital receiving in those months 230 and 200 patients respectively; 117 horses died and 15 were killed as incurable. Only 3 cases of glanders were brought in, all of them being killed; 178 horses were treated for influenza; 8 cases of tetanus were observed, of which 5 died.

Hæmoglobinuria (Azoturia) occurred in 5 horses; 3 were cured and 2 died. Analysis of the urine gave the following results:

Case 1. Died on the second day. Quantity of urine, 5.5 liters. Sp. grav., 1.012. Reaction, acid. Albumen, large quantity. Chlorides, lessened. Phosphates, lessened. Urates, 6.65 per cent. in liter of urine.

CASE II.

| | 1st day. 4.5 liter urine. | 2d day. 9 liters. | 3d day. Urine passed naturally. |
|---|---|---|---|
| Sp. grav., | 1040 | 1041 | 1040 |
| Reaction, | acid. | acid. | acid. |
| Albumen, | large quantity. | some. | traces of. |
| Chlorides, | " " | lessened. | much. |
| Phosphates, | decreased. | decreased. | decreased. |
| Urates, | 21.97 in liter urine. | 26.74. | 18.85. |

The others were about the same in all; the quantity of albumen *was very great.*

Pericarditis in an 8-year-old gelding. Status fraeseus at time of admission. Temperature, 39.7° C. Respirations, 24 per minute; *the entire thorax being moved and the abdominal muscles powerfully contracted in each respiration.* Percussion of thorax gave resonance in all parts. The heart dullness increased in a retro-superior direction from its normal position. Pulse intermittent, weak and accelerated. Constipation. Urine free from albumen. Appetite poor.

Therapeutics. Tinct. Digitalis, Glauber Salts and Salicylate of Soda, with cold water packs. Patient died.

In the surgical clinic for large animals there were treated 810, of which 804 were horses; 1459 dogs were admitted to the dog hospital; 43 dogs were admitted on account of rabies, 24 having the violent and 9 the still form: while 10 were entered as suspects—of which 4 became confirmed. Nine hundred and seventy-three dogs and four cats were killed for various causes.

In the Veterinary Police District, which is under the control of the school, 3 cases only of glanders were discovered.

Sarcoptes Mange occurred in 3 horses.

Foot and mouth disease was found in 4 places, numbering in all 355 cattle, of which 86 were afflicted—all recovered.

Five hundred and forty-two horses were examined for forensic attestation.

The Report is very interesting in detail, but we have only given some of the essential points.

